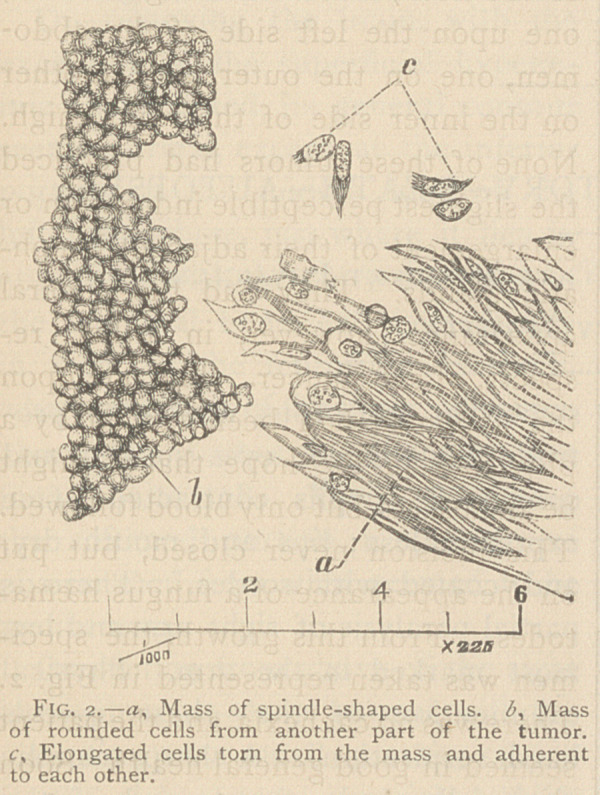# The Difference between Carcinoma and Sarcoma

**Published:** 1875-03-15

**Authors:** 


### MEDICAL EXAMINER.

A

*Semi-Monthly Journal of Medical Sciences.*

EDITED BY N. S. DAVIS, M.D., AND F. H. DAVIS, M.D.

No. VI. CHICAGO, MARCH 15, 1875. VOL. XVI.

### Original Communications.

### THE DIFFERENCE BETWEEN CARCINOMA AND SARCOMA.

THE following cases and illustrations were laid before the Society of Physicians and Surgeons, by Dr. E. Andrews, Professor of Surgery in Chicago Medical College, and Dr. L. Curtis, Professor of Pathology in the Woman's Medical College. As the distinction between these diseases is obscure in many minds, the authors have reproduced the essay in these pages, to give a clear idea of the differential diagnosis.

CASE I. — CARCINOMA. — *Clinical History.* — Mrs. A., of Wisconsin, aged about forty years, presented herself with a small tumor on the foot below the inner malleolus. The mass was of the size of a large hazel-nut, and had been some six months in growing. It was devoid of cuticle; and appeared like a fungus hæmotodes, except that there was little or no bloody effusion. The lymphatic glands were not perceptibly implicated at that time, but a section of the tumor placed under the microscope, showed very clearly the structure of carcinoma, or true cancer.

Ether having been administered, the tumor was excised. Locally, it was a cure; the disease never re-appearing in the original locality; however, in the course of a year or more afterwards, the lymphatic glands above began to show cancerous enlargement. About twenty-one months after the operation, we learn that she was attacked with violent pains in the head, and died in two or three days. No *post-mortem* examination has been reported to us.

*Microscopic Examination.*—Fig. 1 is an exact reproduction of a specimen of this tumor, as prepared by hardening it for some days in dilute chromic acid, and then shaving off very thin slices. The latter were steeped a few minutes in a solution of aniline, and then mounted on a glass slide. The characteristic structure of carcinoma or true cancer is very distinct. The framework of the mass consists of bundles (*trabecula*) of white fibres, surrounding rather large spaces or *alveoli*, as the pathologists term them; which, though varying much in size, average not very far from $\frac{1}{200}$ of an inch in diameter. These *alveoli* are packed full of rounded cells, which, however, do not adhere firmly to the walls of the cavities, nor to each other. Hence, in the thinner parts of the slice they may be brushed out, so as to leave only the fibrous framework, which is well shown in the engraving. This specimen is a soft cancer; but in hard cancers, the bundles of fibres are thicker. These *alveoli* packed full of rounded cells, constitute the essential microscopic character by which we distinguish carcinoma, or true cancer, from all other tumors.

FIG. 1.—*a*, Fibrous framework. *b*, Alveoli, packed full of rounded cells. *c*, *c*, *c*, Alveoli, from which the cells have been partly brushed out.

CASE II. — SARCOMA. — *Clinical History.*—Miss B., aged twenty years, resided in Chicago. Six months ago, she presented herself to a surgeon with a moderate sized tumor in the subcutaneous tissue of the neck, and another of larger dimensions on the inner side of the right thigh. The surgeon consulted, removed the tumors, but after a moderate period, both returned in the same places. Six months later, she fell under our observation. She had now five tumors well defined, besides two or three small nodules, which seemed likely to produce similar growths. None of them were so situated that they could have been derived in the usual course from each other by transmission of germs along the lymphatics, but all were in the subcutaneous connective tissue. One was upon the right side of the neck, one on the right breast, one upon the left side of the abdomen, one on the outer, and another on the inner side of the right thigh. None of these tumors had produced the slightest perceptible induration or enlargement of their adjacent lymphatic glands. They had the general appearance, however, in other respects, of soft cancer. The one upon the abdomen had been lanced by a physician, in the hope that it might be an abscess, but only blood followed. This incision never closed, but put on the appearance of a fungus hæmatodes. From this growth, the specimen was taken represented in Fig. 2. There was no cachexia, and the patient seemed in good general health. Soon afterwards, however, the open ulcer on the abdomen gave origin to a malignant attack of erysipelas, which extending down to the groin, followed the connective tissue under Poupart's ligament, reached the interior, and produced a fatal peritonitis.

*Microscopic Examination.*—Specimens from this tumor are shown in Fig. 2. Part of the growth was what is called round-celled sarcoma, while another portion shaded off towards fibrous structure. There were no alveolar spaces, packed with loose cells, but some parts consisted of pure cells, while others were made up of adherent fusiform cells, shading off in places into a solid fibrous structure. Thus, though the mass contained both cells and fibres, yet the fibres enclosed no *alveoli*, and constituted no framework. Where fibres existed, they constituted the entire mass; and where round or fusiform cells prevailed, they, in like manner, constituted the entire structure, excepting, of course, that occasional blood-vessels penetrated the tissue.

FIG. 2.—*a*, Mass of spindle-shaped cells. *b*, Mass of rounded cells from another part of the tumor. *c*, Elongated cells torn from the mass and adherent to each other.

*Comparison of the Pathology.*—If now we compare these diseases, we shall see an apparent reason for their different clinical history.

Pathologists at present are of the opinion that the *alveoli* of carcinoma are enlarged lymphatic spaces, and lined with the lymphatic endothelium. If this is so, the loosely connected cancer cells in them are inside the lymphatic system, and hence would naturally transmit germs along the lymphatic trunks to be developed wherever they might be arrested, which would usually be at the nearest lymphatic glands. This, if it shall be finally established, will fully explain the infection, and the almost exclusive multiplication of carcinomatous tumors along the routes of the lymphatic system.

On the other hand, the cells or fibres of sarcoma are judged by pathologists to be outside of the lymphatic cavities, and hence they transmit no germs along those routes, and do not infect the lymphatic glands.

As the cells of sarcoma are firmly adherent to each other, they are not easily dislodged and distributed by any cause, hence sarcoma is only occasionally found multiple.

The following tabular view will give a condensed and clear idea of the difference between the two diseases:

CLINICAL HISTORY.

| CARCINOMA. | SARCOMA. |
|---|---|
| The tumors generally multiply, and do so almost exclusively along the lymphatic routes; hence, after complete excision of the original tumor, there are usually contaminated lymphatic glands left which become carcinomatous tumors. | The tumors rarely multiply, and when they do it is almost never by the lymphatic routes; hence, after complete excision, there is considerable hope of permanent exemption from the disease. |

MICROSCOPIC STRUCTURE.

| | |
|---|---|
| The tumor consists of a fibrous framework, surrounding *alveoli*, or cavities averaging about 1-200 of an inch in diameter, which cavities are packed full of nucleated cells, rather larger than blood corpuscles. | The tumor consists either of rounded or spindle-shaped cells, which latter often assume the form of fibres, but the fibres do not enclose cavities filled with cells. In one region of the tumor, the cells, if they exist, constitute the whole tissue; or in another part, if fibres are found, the whole mass consists of fibres. |

| | |
|---|---|
| The cells are not adherent to each other, and hence can be easily brushed out of their specimens, leaving the fibrous framework empty. | The cells are adherent to each other, separating with difficulty. |
| The cells are either rounded, or else irregular like those of epithelium. | The cells may be rounded, but are often spindle-shaped, or even prolonged into fibres. |
| It is believed that the alveoli are dilated lymphatics, and consequently that the contained cells are inside the lymphatic system. | It is believed that the cells are outside of the lymphatic cavities. |

*Practical Conclusions.*—Carcinoma and sarcoma are probably both local diseases in the outset, and could they be excised or otherwise destroyed sufficiently early, would result in a permanent cure. The fact is, however, that in carcinoma germs have usually been sent along the lymphatics, beyond the reach of the surgeon, long before the patient presents himself for treatment; hence, the removal of the original tumor does not prevent the recurrence in the lymphatic glands. The cancer, however, ought to be removed, and the earlier the better; and it would seem to be our duty at the same time to remove the corresponding lymphatic glands, even if they are not perceptibly enlarged, because their infection may be considered as already accomplished, though the development be not yet manifest. There is no rational doubt that early and thorough extirpation of cancer on the average prolongs life and comfort.

The removal of sarcoma is a much more hopeful procedure. The glands not being infected, the patient often shows little tendency to a recurrence after a thorough excision, so that an early and complete removal is above all things to be desired.

No. 6 Sixteenth St., Chicago.

## Figures and Tables

**Fig. 1. f1:**
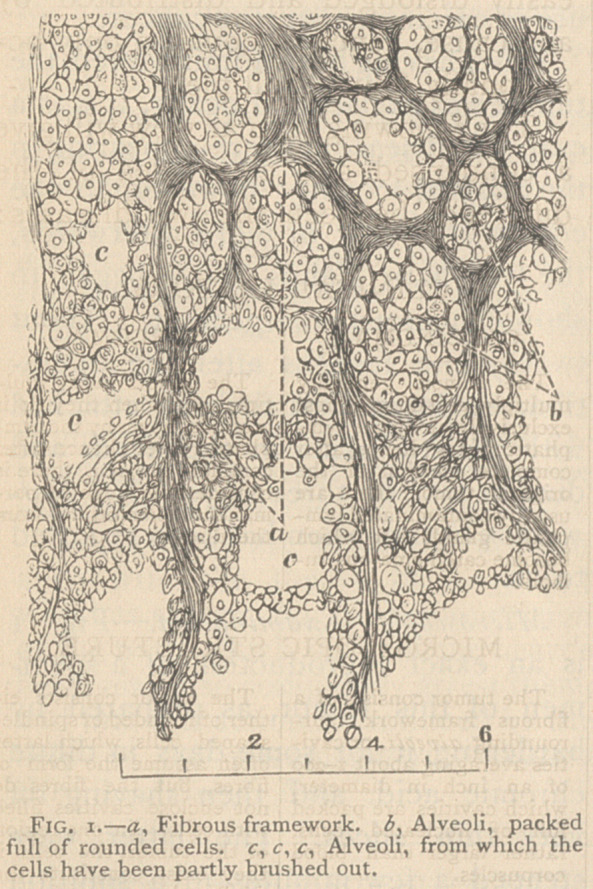


**Fig. 2. f2:**